# Supplementary material for: Study on the Adsorption Properties of Graphene Oxide/Laponite RD/Chitosan Composites
Source: Materials (Basel). 2021 Jun 11;14(12):3224. doi: 10.3390/ma14123224 (PMC8230705; doi:10.3390/ma14123224)
Supplement: Supplementary file 1 [file materials-14-03224-s001.zip › materials-1228698-supplementary.pdf]

Supporting Information

Thermogravimetric Analysis

The thermogravimetric analysis curve of 30% doping amount is given in Figure S1. There are also mainly two decomposition temperatures in the range of room temperature to 500 °C, just like the previous curve. The first weight loss stage occurs in the range of room temperature to 100 °C, mainly due to the evaporation of residual water on the surface or inside of the sample. The second weight loss occurred in the range of 300~350 °C, mainly due to the decomposition of chitosan and the fracture of aerogel molecular chains. It's worth noting that the second decomposition temperature of curve F is more concentrated. It can be concluded that the thermal stability is gradually enhanced with the increase of the content of composite.

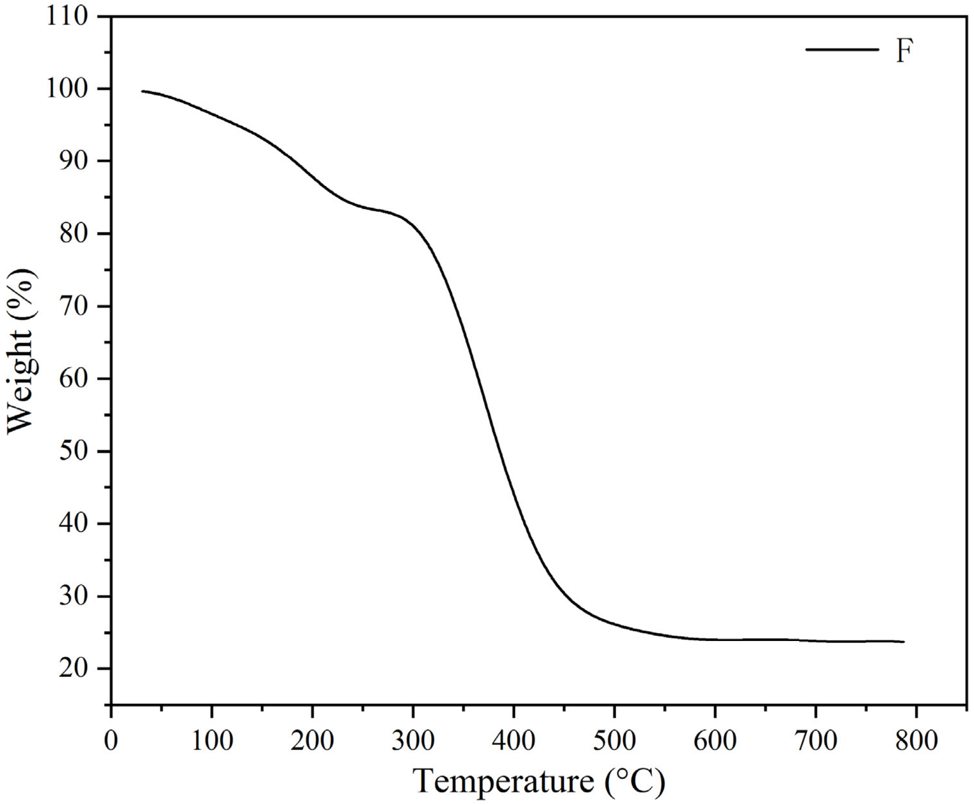

Figure S1. TG curve of hybrid aerogel with doping amount 30% (F).

Density

The true density of the sample (AP-RD/GO-CT (20%)) was measured by the specific surface area analyzer (V-Sorb 2800P, Beijing). Information related to true density were obtained from the lab report. And the true density is 0.0216 g/cm^3 under the pressure of 0.1 MPa and the temperature is 25 °C. In terms of true density, this sample does not belong to ultra-low density aerogel. For example, low density (8.1 kg/m^3) and high porosity aerogel based on nanofibrillated cellulose have prepared by freeze drying method. And this aerogel shows high cyclic compressibility and comparable thermal conductivity with air. [1] The samples presented in this paper are slightly denser than the ultralow density aerogels reported. [1-2] This may be due to the

fact that the sample has a denser cross-linking network. Besides, the relatively dense network structure also contributes to the adsorption.

Synthesis of Graphene Oxide

GO was prepared by the oxidation of natural graphite powder (325 mesh). Typically, graphite powder (3.0 g) was added to concentrated H_2SO_4 (70 mL) under stirring in an ice bath. Under vigorous agitation, $KMnO_4$ (9.0 g) was added slowly to keep the temperature of the suspension lower than 20 °C. Successively, the reaction system was transferred to a 40 °C oil bath and vigorously stirred for about 0.5 h. Then, 150 mL water was added, and the solution was stirred for 15 min at 95 °C. Additional 500 mL water was added and followed by a slow addition of 15 mL H_2O_2 (30 %), turning the color of the solution from dark brown to yellow. The mixture was filtered and washed with 1:10 HCl aqueous solution (250 mL) to remove metal ions. The resulting solid was dried in air and diluted to 600 mL, making a graphite oxide aqueous dispersion. Finally, it was purified by dialysis for one week using a dialysis membrane with a molecular weight cut off of 8000–14,000 g•mol^{-1} to remove the remaining metal species. The resultant graphite oxide aqueous dispersion was then diluted to 1.2 L, stirred overnight and sonicated for 30 min to exfoliate it to GO [3].

References

1. Gupta P, Singh B, Agrawal A K, et al. Low density and high strength nanofibrillated cellulose aerogel for thermal insulation application[J].

Materials & Design, 2018, 158: 224-236

2. Arndt E M, Gawryla M D, Schiraldi D A. Elastic, low density epoxy/clay aerogel composites[J]. Journal of Materials Chemistry, 2007, 17(33): 3525-3529.
3. Chen J, Yao B, Li C, et al. An improved Hummers method for eco-friendly synthesis of graphene oxide[J]. Carbon, 2013, 64: 225-229.
